# Association of Asthma Control With Caregivers' Knowledge and Practices for Children With Asthma in the Tabuk Region of Saudi Arabia: A Cross-Sectional Study

**DOI:** 10.7759/cureus.35162

**Published:** 2023-02-18

**Authors:** Badr Alsayed, Abeer Alatawi, Omniyyah M Alatawi, Rawan H Alatawi, Asmaa M Alfuhaymani, Jomanah K Aljohani

**Affiliations:** 1 Internal Medicine, University of Tabuk, Tabuk, SAU; 2 Nursing, University of Tabuk, Tabuk, SAU

**Keywords:** caregiver, practices, knowledge, asthma control test, asthma

## Abstract

Introduction

Asthma is a chronic heterogeneous inflammatory disease that affects millions of individuals around the globe. Standardized asthma management is crucial to maintain and control the disease. Caregivers are the leading players in managing asthma during childhood. Studies are lacking in the Tabuk region. The aim of this study was to elucidate knowledge levels and common practices of caregivers of pediatric patients with asthma in the Tabuk region of Saudi Arabia and its impact on asthma control.

Methodology

A validated cross-sectional survey of the population of the Tabuk region was conducted from July 1, 2022, to September 30, 2022. Convenience sampling via an internet-based questionnaire within the study area was deployed, followed by phone interviews.

Results

A total of 393 caregivers completed the questionnaire, and the data were analyzed. The median age of asthmatic children was nine years, and most of them were males (60.8%). Most caregivers had a higher education (62.1%). The symptoms of allergic rhinitis were found in almost 80% of children. Pulmonary function tests were performed in 42.5% of children, and only one-third underwent radioallergosorbent (RAST) skin testing. About half the children had an asthmatic attack and an emergency department visit once during the previous 12 months, and most were hospitalized during that period. Most caregivers showed good knowledge (score=7) about symptoms of asthma and factors that could worsen the child's asthma, as well as good asthma control practices (score ≥7). Children with poorly controlled asthma were younger, had significant allergic rhinitis symptoms (30%), and underwent pulmonary function tests (60%).

Conclusion

In the Tabuk region, the extent of asthma control was significantly associated with caregivers' knowledge and practices for children with asthma. Future public education campaigns should focus on closing the observed knowledge and practice gaps to reduce the impact of childhood asthma.

## Introduction

Asthma is a chronic heterogeneous inflammatory disease that affects the airways; 1%-18% of children and adults in various communities present with this condition. Airway hyperresponsiveness is an exaggerated airway-narrowing and it occurs as a result of exposure to specific triggers, including viruses, allergens, and exercise. Furthermore, asthma is associated with airway inflammation. The diagnosis of asthma can be confirmed through the following symptoms: variable expiratory airflow limitations in addition to common respiratory manifestations, including wheezing, shortness of breath, tightness of the chest, and a cough that changes progressively in severity. According to Serebrisky et al. [1] and Alqahtani et al. [2], exercise, allergen or irritant exposure, weather changes, or respiratory viral infections are some triggers of asthma. A national Saudi domestic investigation of chronic medical conditions, i.e., asthma, performed by Moradi-Lakeh et al. [3], stated that self-reported asthma had a prevalence of 4%. Furthermore, physician-diagnosed asthma among young Saudi adults living in the capital was reported to have a prevalence of 11.3%. According to Al Ghobain et al. [4], this value increased over two decades, and more than a third of these patients had uncontrolled asthma.

Furthermore, regarding disease control, an epidemiological study by Al-Jahdali et al. [5] found that only 30.1% had controlled asthma among 1009 asthmatic patients in Saudi Arabia. According to Alhammad et al. [6], controlling a child's asthma may be influenced by the level of parental knowledge and practices, and controlling a child's asthma is primarily the parent's responsibility; parents need to be educated on the causes and symptoms of asthma, medications, and equipment available, self-management approaches, triggers of asthma, and preventive measures [7]. Therefore, researchers have designed numerous questionnaires to evaluate the knowledge and practices related to parental asthma. Due to its importance, the Saudi Initiative for Asthma expert panel on asthma management in children has included "patient/caregiver assessment for control" as an integral part of asthma control assessment, as reported by Al-Moamary et al. [8]. Male sex, higher education, and fixed combination therapy were positive predictive factors of asthma control in an epidemiological study by Al-Jahdali et al. [5]. Saudi Arabia has 13 provinces/regions and a land area of about 2,150,000 km²; studies are lacking in the Tabuk region.

The purpose of the present study was to evaluate the knowledge and practices among parents of children with asthma in the Tabuk region, North-western Saudi Arabia. In addition, the study explored the potential association of the level of parents' awareness and practice with the level of disease control.

## Materials and methods

Study design, setting, and data

A cross-sectional study included adult caregivers of asthmatic children using social media platforms such as WhatsApp, Facebook, and Telegram. The participants comprised of persons from the Tabuk region in Saudi Arabia, and the study was conducted between July 1, 2022, and September 30, 2022. We included caregivers of children (aged >5 and <16 years) diagnosed with asthma by healthcare providers. Children with other chronic lung diseases excluded, e.g., cystic fibrosis, bronchopulmonary dysplasia, and primary ciliary dyskinesia. We also excluded subjects who refused to participate and those with incomplete data.

Sampling and sample size

Convenience sampling via an online-based questionnaire was deployed within the community area, followed by phone interviews to ensure understanding and the accuracy of participants' answers. The survey was open to all. No personal information was collected during the survey, and the data were collected by fifteen trained data collectors who knew the study goals. Calculations to determine the sample size using the Raosoft website revealed that a sample size of 377 based on a 5% margin error, 95% confidence level, and 50% response rate would be ideal.

Study instrument and data collection

Data collection was carried out using a structured questionnaire including demographic information, such as age, sex, educational level, marital status, employment, residence, and nationality. The questions were designed to evaluate the level of knowledge and practices of participants related to asthma and were adopted under the Creative Commons Attribution (CC BY) license from a validated data collection tool by Fasola et al. [9]. Based on focus group discussion and expert opinions, and considering Saudi cultural norms and the study setting, the tool was slightly modified regarding the following questions: mold in the child's bedroom, spirometry readings, birth order status, and receiving an asthma action plan from the treating physician.

Ten healthcare specialists validated the questionnaire's content, which was then piloted among 40 people who were caregivers of asthmatic children. The questionnaire was subsequently converted to Arabic to let people respond in their own language, considering the accuracy and clarity of English to Arabic language translation to ensure validity, according to Global Initiative for Asthma Main Report [10]. After the piloting, the final version of the 38-item questionnaire was thoroughly explained to 15 senior medical students who interviewed the caregivers and collected the data. Assessment of the asthma control level was carried out with the Asthma Control Test (ACT) according to the Global Initiative for Asthma guidelines [11].

Ethical considerations

Approval for the study protocol was obtained from the Tabuk Institutional Review Board (protocol no. TU-077/022/152). Investigators secured the data and confirmed that the data were not used for any purpose outside the study. Personal information (e.g., name and contact information) was not recorded in the data entry software to ensure participant anonymity. All participants were individually assigned unique identifier codes. Participants were informed about the study objectives, methodology, risks, and benefits. Filling out the questionnaire was considered consent to participate in the study.

Statistical analysis

The Statistical Package for Social Sciences version 26 for Windows (IBM Inc., Armonk, New York) was used for statistical analyses. Numerical variables were not normally distributed and are summarized as the median and interquartile range (IQR). Intergroup comparisons were performed using the Kruskal-Wallis test; if the data were significant, the post hoc Dunn-Bonferroni test was performed. Categorical variables have been included in the form of counts and percentages. The association between the categorical variables and asthma control was evaluated via the Pearson Chi-squared and Fisher-Freeman-Halton exact tests. A p-value of <0.05 indicated statistical significance.

## Results

Of 537 questionnaires, 393 were completed (response rate of 73.18%). The included children were aged 6-16 years (median age nine years). The median ages at the onset of asthma symptoms and diagnosis were four and five years, respectively. Most children were males (60.8%). Half the children were third to fifth in order among their siblings. The mother was the caregiver among 88.3% of the children. Most caregivers had a higher education (62.1%). About one-quarter of the children had pets in their houses, and exposure to tobacco smoke was noted in about half the cases. Only one-third of the children had a radioallergosorbent (RAST) test performed to identify the allergens to which they were sensitized. Symptoms of allergic rhinitis were intermittent (67.9%) or persistent (9.7%) in most children. About one-fifth of children received no asthma treatment, 67.7% received treatment when needed, and 14% had regular treatment at least every three months. Pulmonary function tests were performed in 42.5% of children. About half the children had an asthmatic attack and an emergency department (ED) visit once within the previous 12 months, and most children were hospitalized once (38.9%) or more than once (19.3%) during this period (Table 1).

**Table 1:** Characteristics of the studied children with asthma, their caregivers, and their environments (n=393) Values have been included in the form of median {interquartile range} (minimum - maximum) or as numbers (percentage)

Characteristics of children, caregivers, and the environment		Total number = 393
Child's age (years)		9 {6-11} (6-16)
Gender	Female	154 (39.2%)
	Male	239 (60.8%)
Age at the onset of asthma symptoms, years		4 {2-6} (1-14)
Age at which asthma was diagnosed, years		5 {3-7} (1-16)
Order among the siblings		3 {2-5} (1-13)
Weight (kg)		32 {24-40} (8-100)
Height (cm)		130 {115-144} (56-168)
Body mass index (kg/m^2^)		19 {16-21} (7-70)
Caregiver	Mother	347 (88.3%)
	Others	46 (11.7%)
Education level	Upper secondary	149 (37.9%)
	Higher education	244 (62.1%)
Pet(s) at the patients' (dog, cat, or birds)	No	297 (75.6%)
	Yes	96 (24.4%)
Environmental tobacco smoke	No	203 (51.7%)
	Yes	190 (48.3%)
Number of family members in the same house		6 {5-8} (2-22)
Number of rooms in the house		5 {4–6} (2–20)
Have you ever conducted a RAST test to discover if your child is sensitized to which allergens?	No	175 (44.5%)
	Yes	142 (36.1%)
	I don’t know	76 (19.3%)
Allergic rhinitis symptoms (sneezing, runny nose, itchy nose, and eye)	No	88 (22.4%)
	Intermittent	267 (67.9%)
	Persistent	38 (9.7%)
Asthma treatment	No treatment	72 (18.3%)
	As required	266 (67.7%)
	Regular for minimum 3 months	55 (14.0%)
Did your child undergo a pulmonary function test?	No	226 (57.5%)
	Yes	167 (42.5%)
Asthma attack in the previous 12 months	None	113 (28.8%)
	Once	187 (47.6%)
	More than once	93 (23.7%)
ED visits, previous 12 months	None	100 (25.4%)
	Once	182 (46.3%)
	More than once	111 (28.2%)
Hospitalizations, previous 12 months	None	164 (41.7%)
	Once	153 (38.9%)
	More than once	76 (19.3%)

Most caregivers showed good knowledge about asthma symptoms and factors that could worsen the child's asthma. However, the knowledge of most parents was inaccurate (79.1%) regarding repeated coughing as an indicator of an asthmatic attack. The total knowledge score was satisfactory in most parents, with about half the caregivers scoring ≥7 (out of a maximum score of nine). Concerning caregiver practices, most participants reported good practices regarding avoiding child exposure to tobacco smoke (83.5%), use of inhaled beta-2 agonists during an acute attack (84.2%), calling the doctor during an acute attack (71.0%), going to the ED (90.1%), receiving an action plan from the child's doctor (73.3%), and willingness to follow the plan (88.8%). The points with less adherence to best practices included avoiding fluffy toys, use of oral corticosteroids, and antibiotic use during an acute asthma attack. The total practice score was good, with half the caregivers scoring ≥7 (out of a maximal score of 10; Table 2).

**Table 2:** Knowledge of the children's caregivers about the management of asthma and their practices (n=393) Values have been presented as median {interquartile range} (minimum - maximum) or as numbers (percentage)

Knowledge	Incorrect	Correct
Can stopping medications worsen your child's asthma?	117 (29.8%)	276 (70.2%)
Does your child's asthma worsen with a cold?	112 (28.5%)	281 (71.5%)
Does allergen exposure worsen your child's asthma?	53 (13.5%)	340 (86.5%)
Does your child's asthma worsen with exposure to cold air?	116 (29.5%)	277 (70.5%)
Is your child’s asthma worsened by exposure to traffic?	85 (21.6%)	308 (78.4%)
Can exposure to tobacco smoke worsen your child’s asthma?	47 (12.0%)	346 (88.0%)
Does repeated coughing indicate the onset of an asthma attack?	311 (79.1%)	82 (20.9%)
Is asthma indicated by chest tightness?	94 (23.9%)	299 (76.1%)
Does a dry cough after exercise indicate an asthma attack?	119 (30.3%)	274 (69.7%)
Knowledge score	7 {5-8} (1-9)	
Practice	Poor	Good
Have you prevented your child from being exposed to tobacco smoke after his/her asthma diagnosis?	65 (16.5%)	328 (83.5%)
Have you prevented your child from having fluffy toys after his/her asthma diagnosis?	152 (38.7%)	241 (61.3%)
Will you consider treating your child with oral corticosteroids if he/she has an acute asthma attack?	226 (57.5%)	167 (42.5%)
Will you consider treating your child with inhaled corticosteroids if he/she has an acute asthma attack?	228 (58.0%)	165 (42.0%)
Will you consider treating your child with inhaled beta-2 agonists if he/she has an acute asthma attack?	62 (15.8%)	331 (84.2%)
Will you consider treating your child with antibiotics if he/she has an acute asthma attack?	196 (49.9%)	197 (50.1%)
Will you call the doctor if your child has an acute asthma attack?	114 (29.0%)	279 (71.0%)
Will you take your child to the emergency department in case of an acute asthma attack?	39 (9.9%)	354 (90.1%)
Did your child's doctor give you an asthma action plan?	105 (26.7%)	288 (73.3%)
Will you adhere to the asthma action plan if your child has an acute asthma attack?	44 (11.2%)	349 (88.8%)
Practice score	7 {6-8} (1-10)	

The responses of the asthmatic children and caregivers to the ACT revealed that the asthma of most children was well-controlled (60.3%). In comparison, 27% of cases were partially controlled, and 12.7% were poorly controlled (Table 3).

**Table 3:** Responses to the asthma control test (n=393) Values have been presented as median {interquartile range} (minimum - maximum) or as numbers (percentage)

Asthma control test		Total number = 393
How is your asthma currently?	Very bad	13 (3.3%)
	Bad	27 (6.9%)
	Good	155 (39.4%)
	Very good	198 (50.4%)
To what extent does your asthma affect you when running, exercising, or playing sports?	It's a big problem, and I can't do what I want to do	30 (7.6%)
	It's a problem, and I don't like it	81 (20.6%)
	It's a bit problematic, but it's okay	191 (48.6%)
	It's not a problem	91 (23.2%)
Do you cough because of your asthma?	Yes, all of the time	13 (3.3%)
	Yes, most of the time	75 (19.1%)
	Yes, some of the time	251 (63.9%)
	No, none of the time	54 (13.7%)
Does your asthma wake you up during the night?	Yes, all of the time	12 (3.1%)
	Yes, most of the time	53 (13.5%)
	Yes, some of the time	215 (54.7%)
	No, none of the time	113 (28.8%)
In the past four weeks, for how many days did your child present with symptoms of daytime asthma?	Every day	5 (1.3%)
	19-24 days	11 (2.8%)
	11-18 days	22 (5.6%)
	4-10 days	80 (20.4%)
	1-3 days	157 (39.9%)
	Not at all	118 (30.0%)
In the past four weeks, for how many days did your child experience asthma-related wheezing during the day?	Every day	12 (3.1%)
	19-24 days	6 (1.5%)
	11-18 days	17 (4.3%)
	4-10 days	67 (17.0%)
	1-3 days	137 (34.9%)
	Not at all	154 (39.2%)
In the past 4 weeks, for how many days did your child wake up at night because of asthma?	Every day	8 (2.0%)
	19-24 days	7 (1.8%)
	11-18 days	19 (4.8%)
	4-10 days	58 (14.8%)
	1-3 days	139 (35.4%)
	Not at all	162 (41.2%)
Test score		21 {18-23} (2-27)
Asthma control grading	Very poor control	50 (12.7%)
	Not well-controlled	106 (27.0%)
	Well-controlled	237 (60.3%)

Comparison among the three asthma control grades revealed that the age of the children when attempting the questionnaire appeared to be significantly lower in the poorly controlled group than in the other two groups (p=0.027). The poorly controlled group had a significantly higher percentage of children who had persistent allergic rhinitis (30% vs. 12.3% and 4.2%) and underwent pulmonary function tests (60% vs. 43.4% and 38.4%). Moreover, a significantly higher percentage of this group received regular treatment for at least three months (36.0% vs. 19.8% and 6.8%). Male children were more prevalent in the well-controlled group; however, this difference was non-significant (p=0.072). Patients in the poorly controlled group had a tendency to be relatively younger at the onset of asthma symptoms and its diagnosis; however, this value was not statistical significance (p=0.052). Other statistically non-significant tendencies were observed, such as a higher percentage of other caregivers, the presence of pets, and the performance of the RAST test in the poorly controlled group (p>0.05) (Table 4).

**Table 4:** Comparison among the grades of asthma control regarding the characteristics of the children, their caregivers, and the environment (n=393) Values have been presented as median {interquartile range} (minimum - maximum) or as numbers (percentage) * significant at p<0.05; a: Kruskal–Wallis test; b: Pearson's Chi-squared test for independence of observations I: significantly different from the poorly controlled group in the post hoc test; II: significantly different from the not well-controlled group in the post hoc test; III: significantly different from the well-controlled group in the post hoc test

Characteristics of the children, their caregivers, and the environment			Asthma control grading			
			Very poor control (N=50)	Not well-controlled (N=106)	Well-controlled (N=237)	p-value
Child's age (years)		7 {6-10} (6-15) II, III		9 {7-12} (6-16) I	9 {7-12} (6-15) II	0.027*a
Sex	Female	23 (46.0%)		49 (46.2%)	82 (34.6%)	0.072 b
	Male	27 (54.0%)		57 (53.8%)	155 (65.4%)	
Age at asthma symptom onset, years		3 {2-5} (1-10)		4 {2-6} (1-14)	4 {2-6} (1-14)	0.052 a
Age at asthma diagnosis, years		4 {3-6} (1-14)		4 {3-7} (1-16)	5 {3-7} (1-14)	0.102 a
Order among siblings		3 {1-5} (1-13)		3 {2-5} (1-10)	3 {2-4} (1-12)	0.268 a
Weight (kg)		28 {20-35} (12-75)		32 {23-40} (8-100)	33 {25-40} (10-95)	0.103 a
Height (cm)		121 {115–138} (90–157)		130 {120–146} (75–165)	131 {115–144} (56–168)	0.132 a
Body mass index (kg/m^2^)		18 {16–20} (8–37)		19 {16–22} (7–39)	19 {16–21} (10–70)	0.385 a
Care provider	Mother	42 (84.0%)		97 (91.5%)	208 (87.8%)	0.365 b
	Others	8 (16.0%)		9 (8.5%)	29 (12.2%)	
Educational level	Upper secondary	21 (42.0%)		45 (42.5%)	83 (35.0%)	0.346 b
	Higher education	29 (58.0%)		61 (57.5%)	154 (65.0%)	
Pets in the child's home (dog, cat, and bird)			17 (34.0%)	28 (26.4%)	51 (21.5%)	0.150 b
Environmental tobacco smoke			24 (48.0%)	50 (47.2%)	116 (48.9%)	0.954 b
Number of family members in the same house		6 {5-8} (2-15)		6 {5-8} (2-12)	6 {5-7} (2-22)	0.630 a
Number of rooms in the house		5 {4-7} (2-20)		5 {4-6} (2-20)	5 {4-6} (2-18)	0.964 a
Conducted a RAST test to discover to which allergens the child is sensitized			23 (46.0%)	40 (37.7%)	79 (33.3%)	0.460 b
Allergic rhinitis symptoms (sneezing, runny nose, itchy nose, and eye)	No	12 (24.0%)		9 (8.5%)	67 (28.3%)	<0.001*b
	Intermittent	23 (46.0%)		84 (79.2%)	160 (67.5%)	
	Persistent	15 (30.0%)		13 (12.3%)	10 (4.2%)	
Asthma treatment	No treatment	5 (10.0%)		11 (10.4%)	56 (23.6%)	<0.001*b
	As needed	27 (54.0%)		74 (69.8%)	165 (69.6%)	
	Regular for at least 3 months	18 (36.0%)		21 (19.8%)	16 (6.8%)	
Pulmonary function test performed for the child			30 (60.0%)	46 (43.4%)	91 (38.4%)	0.019*b

The percentage of caregivers with good knowledge about asthma symptoms and the worsening factors showed a tendency to increase with the level of asthma control. However, statistical significance was only reached regarding their knowledge about the worsening effect of stopping asthma medications. Likewise, the percentage of caregivers with good asthma management practices showed a similar tendency, with statistical significance regarding the use of inhaled corticosteroids and beta-2 agonists, and avoiding antibiotics (p=0.029, 0.047, and 0.026, respectively; Table 5).

**Table 5:** Comparison among the grades of asthma control regarding the knowledge and practices of caregivers concerning their children's asthma (n=393) Values are presented as number (percentage) * significant at p<0.05; a: Pearson's Chi-squared test for independence of observations

Knowledge	Asthma control grading			
	Very poor control (N=50)	Not well-controlled (N=106)	Well-controlled (N=237)	p-value a
Does stopping the drugs worsen your child's asthma?	40 (14.5%)	88 (31.9%)	148 (53.6%)	<0.001*
Does your child's asthma worsen with a cold?	36 (12.8%)	84 (29.9%)	161 (57.3%)	0.100
Does allergen exposure worsen your child's asthma?	44 (12.9%)	97 (28.5%)	199 (58.5%)	0.159
Does your child’s asthma worsen with exposure to cold air?	34 (12.3%)	81 (29.2%)	162 (58.5%)	0.293
Is your child’s asthma worsened by exposure to traffic?	37 (12.0%)	89 (28.9%)	182 (59.1%)	0.239
Can exposure to tobacco smoke worsen your child’s asthma?	43 (12.4%)	97 (28.0%)	206 (59.5%)	0.429
Does repeated coughing indicate the onset of an asthma attack?	11 (13.4%)	19 (23.2%)	52 (63.4%)	0.684
Does chest tightness indicate the onset of an asthma attack?	38 (12.7%)	85 (28.4%)	176 (58.9%)	0.493
Does a dry cough after exercise indicate the onset an asthma attack?	35 (12.8%)	77 (28.1%)	162 (59.1%)	0.726
Practice				
Have you prevented your child from being exposed to tobacco smoke after his/her asthma diagnosis?	40 (12.2%)	83 (25.3%)	205 (62.5%)	0.131
Have you prevented your child from having fluffy toys after his/her asthma diagnosis?	29 (12.0%)	62 (25.7%)	150 (62.2%)	0.613
Will you consider treating your child with oral corticosteroids if he/she has an acute asthma attack?	25 (15.0%)	50 (29.9%)	92 (55.1%)	0.182
Will you consider treating your child with inhaled corticosteroids if he/she has an acute asthma attack	28 (17.0%)	49 (29.7%)	88 (53.3%)	0.029*
Will you consider treating your child with inhaled beta-2 agonists if he/she has an acute asthma attack	44 (13.3%)	96 (29.0%)	191 (57.7%)	0.047*
Will you consider treating your child with antibiotics if he/she has an acute asthma attack?	17 (8.7%)	50 (25.5%)	129 (65.8%)	0.026*
Will you call the doctor if your child has an acute asthma attack?	31 (11.1%)	76 (27.2%)	172 (61.6%)	0.320
Will you take your child to the emergency department in case of an acute asthma attack?	40 (11.3%)	98 (27.7%)	216 (61.0%)	0.050
Did your child's doctor give you an asthma action plan?	38 (13.2%)	78 (27.1%)	172 (59.7%)	0.881
Will you adhere to the asthma action plan if your child has an acute asthma attack?	42 (12.0%)	99 (28.4%)	208 (59.6%)	0.160

## Discussion

Bronchial asthma is a major public health concern worldwide. as reported by Serebrisky et al. [1]. According to Alqahtani et al. [2], this condition is considered a significant socioeconomic problem, as poor control can result in repeated hospital admissions, school absences, and fatalities in severe cases. Parents are majorly responsible for the care provision and management of their children's asthma. Several studies by Licari et al. [7], Alhammad et al. [6], and Zhao et al. [12] reported that parents' knowledge related to the triggers of asthma, preventive measures, and medications is crucial, and their practices are vital to achieving good asthma control.

In the present study, we examined the knowledge and practices of the parents/caregivers of children with asthma in the Tabuk region of Saudi Arabia. In addition, the study explored the potential association of the level of parents' awareness and practice with the level of disease control. A total of 393 parents (out of 537) consented to the study and completed the questionnaires.

Asthmatic children

The children's characteristics were similar to those in previous studies by Fasola et al. [9] and Roncada et al. [13]. According to the report by Roncada et al. [13], the mean ages of children with asthma at diagnosis and at the study time were approximately six years and 10 years, respectively, and 47% were males. Fasola et al. [9] reported that male children were predominant in their study (65.5%; mean age, 9.1 years). In the current study, approximately half the children had an asthmatic attack and an ED visit once during the previous 12 months. Most children were hospitalized during the previous 12 months, either once (38.9%) or more than once (19.3%). These rates are different from those reported by previous studies. In China, Zhao et al. [12] found that 26.8% of asthmatic children visited the ED, and 16.2% were hospitalized during the past 12 months. Fasola et al. [9] reported a higher rate of asthma attacks in the previous 12 months (76.7% of the children) but a lower rate of ED visits and hospitalizations (37.9% and 19.9% of children, respectively). The variations in reported rates may be attributed to differences in the knowledge and practices of the caregivers of children with asthma. Higher knowledge may enable caregivers to manage acute asthma attacks effectively, negating the need for ED visits and hospitalizations.

In the current study, most children (60.3%) had well-controlled asthma, whereas 27% were not well-controlled, and 12.7% were poorly controlled. Other studies reported different rates. In Saudi Arabia, BinSaeed [14] and Al-Zalabani et al. [15] reported that uncontrolled asthma accounted for 59.3% and 62.6% of their series, respectively. However, these studies were conducted in different regions from Tabuk (Riyadh, Al-Madinah Al-Munawarah). Roncada et al. [13] found that asthma was mildly persistent in 37.1% of children, moderately persistent in 38.7%, and severely persistent in 24.2%. In their study, Fasola et al. [9] noted that approximately one-third of the children presented with intermittent asthma, and another third had fully controlled asthma. The poorly controlled group in this study were significantly younger at the time of the study. The ages at the onset of symptoms and diagnosis also tended to be lower in this group. Furthermore, the report by Mirabelli et al. [16] supported these findings and reported that a younger age at onset was associated with an increase in the frequency of asthma-related ED visits and hospitalizations. The poorly controlled group in the present study also exhibited a significantly higher prevalence of persistent allergic rhinitis.

Typically, allergic rhinitis indicates the presence of bronchial asthma and may interfere with asthma control, as reported by Compalati et al. [17] and Kakaje et al. [18]. In addition, a slightly higher percentage of the poorly controlled group owned pets in houses. In Saudi Arabia, Alatawi and Alanazi [19] found that most mothers (67.7%) perceived environmental factors as barriers that impacted the control of their children's asthma. These findings emphasize the role of controlling environmental factors in the management of asthma and warrant the highlighting of environmental factors within future awareness programs. Interestingly, a higher percentage of the poorly controlled group underwent pulmonary function tests and the RAST test, apart from receiving regular treatment for at least three months. However, this may be the result of poor asthma control as parents seek to identify sensitizers, assess the child's health status, and adhere to medications more than the parents whose children have very mild symptoms.

Caregivers of asthmatic children

In the current study, about 18% of caregivers gave no asthma treatment to their children. More than two-thirds of those caregivers had apparently stopped medications as the asthma symptoms were well-controlled. However, about 23% of those receiving no treatment had poorly or inadequately controlled asthma. Barriers that hinder caregivers from adhering to treatment may include inaccessibility of the medications and misconceptions about the drug's effect. The present study did not thoroughly explore parents' misconceptions about asthma medications, but previous studies by Alatawi [19] and Zhao et al. [20] reported various misconceptions, such as fear of drug dependence and adverse effects. The knowledge and practices of caregivers can significantly impact asthma management and control in children. In the present study, parents exhibited an overall satisfactory knowledge regarding the worsening factors of asthma. The higher education of most caregivers in this study could explain this outcome. Additionally, Roncada et al. [13] reported that parents of asthmatic children receive ample instructions and explanations from the treating doctors during their visits to the clinics and ED. Our results were consistent with those of previous studies by Fasola et al. [9], BinSaeed [14], Urrutia-Pereira et al. [21], AlOtaibi et al. [22], Rastogi et al. [23] regarding satisfactory caregiver awareness of the worsening effects of stopping asthma medications, having respiratory infections, and exposure to smoke and allergens.

As reported previously by Fasola et al. [9], Al-Binali et al. [24], Abu-Shaheen et al. [25], most caregivers in the current study (69.7%-76.1%) recognized indicators of asthmatic attacks. However, most respondents did not recognize the vital nature of repeated coughing. The percentage of correct responses about bronchial asthma tended to increase with the level of asthma control, but statistical significance was elicited only in the responses about the effect of stopping asthma medications. Earlier studies found a significant association between parental knowledge and asthma control [6,9,12,14,15]. However, studies by Divecha [26] and Silva et al. [27] reported a lack of a significant association.

Silva et al. [27] showed that the disparity between the studies can be attributed to differences in the questionnaires that assessed knowledge as well as the tendency of some caregivers with defective knowledge to choose milder grades of asthma severity for their children. The overall practices of the caregivers in managing the child's asthma were good in the current study. The most reported good practices included avoiding exposure to tobacco smoke, using inhaled beta-2 agonists, calling the doctor, and visiting the ED during an acute attack. Moreover, most parents were willing to receive and follow the action plan provided by the child's doctor. However, the caregivers' practices showed less adherence to avoiding fluffy toys as well as using oral/inhaled corticosteroids and antibiotics during an acute asthmatic attack. Likewise, previous questionnaires recorded the prevalence of the good practices of using beta-2 agonists or corticosteroid inhalers as well as contacting the doctor or going to the ED [9,23]. The same misconception about the role of antibiotics in asthma management was reported by Fasola et al. [9], Alharbi et al. [28], and Al-Harbi et al. [29].

Awareness campaigns and instructions by healthcare providers focused on the caregivers of asthmatic children must confirm that antibiotics are not useful in managing acute attacks of asthma unless the symptoms and/or laboratory results support a diagnosis of infection. The outcomes of the present study demonstrated that the prevalence of good practices in asthma management tended to increase with the degree of asthma control. However, statistical significance was found only regarding the use of inhaled corticosteroids and beta-2 agonists and the avoidance of antibiotics. Moreover, according to BinSaeed [14], a significantly high percentage of parents in the uncontrolled asthma group had misconceptions related to inhaled corticosteroids. Noureddin et al. [30] reported a significant association between mothers' practices and the severity of asthma, particularly in terms of the correct use of inhalers and the avoidance of triggering factors.

The present study has various strong points, including a sufficient sample that included only the caregivers of asthmatic children, compared with some studies that assessed the knowledge of the public. Moreover, the study identified the defects in knowledge and caregiver practices so that future awareness campaigns targeting caregivers of asthmatic children can effectively address these issues, which are considered barriers to achieving asthma control. However, the study was performed in only one region of Saudi Arabia. Thus, generalization of the results to other regions or populations may not be feasible. Another limitation is that the questionnaire did not thoroughly explore some factors, such as the caregivers' opinion about the safety of asthma medications and the demonstration of correct inhaler use.

## Conclusions

The knowledge and practices of the caregivers of children with asthma were associated with the degree of disease control. Future public education campaigns should concentrate on filling in the known information gap about the classic signs of an asthma attack. Moreover, the best practices for reducing environmental asthma triggers, such as animal allergies, and avoiding unnecessary antibiotics should be followed.
